# Assessment of the role of intracranial hypertension and stress on hippocampal cell apoptosis and hypothalamic-pituitary dysfunction after TBI

**DOI:** 10.1038/s41598-017-04008-w

**Published:** 2017-06-19

**Authors:** Huajun Tan, Weijian Yang, Chenggang Wu, Baolong Liu, Hao Lu, Hong Wang, Hua Yan

**Affiliations:** 1 Graduate School of Tianjin Medical University, Tianjin, 300070 China; 2 Department of Neurosurgery, Tianjin Huanhu Hospital, Tianjin, 300350 China; 3 Department of Neurosurgery, Second Affiliated Hospital of Tianjin University of Traditional Chinese Medicine, Tianjin, 300151 China; 4 Department of Ultrasonography, Tianjin Huanhu Hospital, Tianjin, 300350 China; 5 Tianjin Key Laboratory of Cerebral Vascular and Neurodegenerative Diseases, Tianjin, 300350 China

## Abstract

In recent years, hypopituitarism caused by traumatic brain injury (TBI) has been explored in many clinical studies; however, few studies have focused on intracranial hypertension and stress caused by TBI. In this study, an intracranial hypertension model, with epidural hematoma as the cause, was used to explore the physiopathological and neuroendocrine changes in the hypothalamic–pituitary axis and hippocampus. The results demonstrated that intracranial hypertension increased the apoptosis rate, caspase-3 levels and proliferating cell nuclear antigen (PCNA) in the hippocampus, hypothalamus, pituitary gland and showed a consistent rate of apoptosis within each group. The apoptosis rates of hippocampus, hypothalamus and pituitary gland were further increased when intracranial pressure (ICP) at 24 hour (h) were still increased. The change rates of apoptosis in hypothalamus and pituitary gland were significantly higher than hippocampus. Moreover, the stress caused by surgery may be a crucial factor in apoptosis. To confirm stress leads to apoptosis in the hypothalamus and pituitary gland, we used rabbits to establish a standard stress model. The results confirmed that stress leads to apoptosis of neuroendocrine cells in the hypothalamus and pituitary gland, moreover, the higher the stress intensity, the higher the apoptosis rate in the hypothalamus and pituitary gland.

## Introduction

TBI is often regarded as a silent epidemic because the issues resulting from TBI are often not immediately visible. Furthermore, TBI patients are not very vocal about their condition^1^. In Europe and the USA, 2.5 million people are affected by TBI, which causes chronic disabilities in over 40% of the affected patients^2^. TBI is expected to become the third largest cause of global disease burden by 2020^3^. TBI is a complex pathophysiological reaction that includes primary damage and secondary insults, for example, hypotension, hypoxia, intracranial hypertension and changes in cerebral blood flow and metabolism^4^. Hypopituitarism is a severe complication induced by TBI that causes affective disorder, cognitive dissonance, and physical symptoms; e.g., depression, anxiety, memory and concentration deficits, and fatigue^5^. In recent years, there has been an increasing amount of strong evidence that suggests that patients with TBI are at considerable risk of dysfunction of the hypothalamic-pituitary axis, and this risk is associated with morbidity and possibly mortality^6–9^. Furthermore, clinical trials have demonstrated that treatment with replacement hormones is convenient and physiologically relevant^9, 10^. According to one report, the incidence of hypothalamic-pituitary dysfunction induced by TBI is 5.4% to 90%^6^. Mechanisms have been suggested regarding the factors of hypopituitarism caused by TBI, including primary injury to the hypothalamic–pituitary axis and/or to its blood supply and secondary damage caused by oxidative stress or compression of the hypothalamic–pituitary structure by cerebral edema, cerebral hemorrhage, and intracranial hypertension^4, 11–14^. Reduction of the blood supply to the axis caused by TBI is the main hypothesis to explain the hypothalamic–pituitary dysfunction, however, this hypothesis does not adequately account for all of the hypothalamic–pituitary dysfunction^13^. In clinical work, all the clinical manifestations of a disease should be explained. Therefore, the high incidence of hypothalamic-pituitary dysfunction should be associated with the most common etiologies, and the incidence of these etiologies should exceed the incidence of hypothalamic-pituitary dysfunction. Moreover, the incidence of increased intracranial pressure and stress in the pathophysiological process surpasses the incidence of hypothalamic-pituitary dysfunction. Therefore, we suspected that intracranial hypertension and stress are the major causes of hypothalamic-pituitary dysfunction.

Experimental studies have been performed with epidural balloon inflation, epidural hematoma, or ischemia models to study the physiological, histopathological and biochemical changes in the brain parenchyma that are caused by brain displacement or intracranial hypertension^15–18^. Although many clinical trials have focused on dysfunction of the hypothalamic-pituitary axis caused by TBI^10, 19–21^, few studies have paid close attention to neuroendocrine and physiopathological alterations of the hypothalamic-pituitary axis induced by TBI in the acute phase. It is interesting to explore the neuroendocrine changes and apoptosis of brain cells in the hypothalamic-pituitary axis that are caused by intracranial hypertension.

Trauma is always accompanied by stress. Restraint is the most widely used method to induce stress, and nociceptive stimuli, such as electric shock, pinching, and hyperalgesia induced by pharmaceuticals, are the most powerful factors used to experimentally induce stress^22^. Furthermore, restraint stress has already been widely used in experiments^23, 24^. Therefore, we used restraint and electric shock to induce acute stress. Moreover, to study the effect of different stress intensities on brain cells, we used a restraint and electric shock model to enhance stress intensity.

We used an experimental epidural hematoma animal model to explore the effects of intracranial hypertension on neuroendocrine changes and apoptosis of brain cells in the hippocampus and hypothalamic–pituitary axis in the acute phase. A stress animal model was used to explore the rate of stress-induced apoptosis in the hypothalamus and pituitary gland.

## Methods

### Animal care and group

All methods were performed in accordance with the relevant guidelines and regulations and approved by the Tianjin Key Laboratory of Cerebral Vascular and Neurodegenerative Diseases, China. Laws and rules were strictly obeyed to protect the animals from abuse. The animals were housed in the Tianjin Key Laboratory of Cerebral Vascular and Neurodegenerative Diseases under a constant room temperature, humidity, and 12 h light cycle, and they were provided with sufficient food and water. Before surgery, the animals were acclimated for 7 days.

A total of seventy-two adult male Sprague-Dawley (SD) rats (grade specific pathogen free; 300–320 g) were purchased from the Experimental Animal Center of the Academy of Military Medical Sciences, China. The rats were randomly divided into six groups: Normal 12 h (n = 12), Normal 24 h (n = 12), Sham 12 h (n = 12), Sham 24 h (n = 12), Intracranial hypertension (IH) 12 h (n = 12), and Intracranial hypertension 24 h (n = 12).

Thirty-six adult male New Zealand rabbits (2.2–2.5 kg) were purchased from the Tianjin AoYi Experimental Animal Breeding Co. Ltd., China. The rabbits were randomly divided into six groups: Normal 12 h (n = 6), Normal 24 h (n = 6), Restraint 12 h (n = 6), Restraint 24 h (n = 6), Restraint + electric shock (R + E) 12 h (n = 6), and Restraint + electric shock (R + E) 24 h (n = 6). The two timepoints, i.e., 12 h and 24 h, served as the endpoints of observation, and stress factors were applied only during the initial 6 h of the experiment.

### Rat epidural hematoma model and intracranial hypertension

A standardized epidural hematoma model was based on previous reports^13, 18, 25^. Briefly, before surgery, general anesthesia was performed with 0.3 ml/100 g chloral hydrate administered intraperitoneally. Then, the rats were shaved in the head area and were fixed in the stereo orientation instrument. The skin was sterilized and incised down to the skull along the biparietal sutures through a single sagittal incision. A small square section of the skull (diameter 4 mm) was thinned to the inner compact bony layer on the right parietal bone 5 mm from the intersection of the sagittal suture and the arcuate suture. This protocol was followed by sampling of 0.3 ml autologous blood from the heart. A needle was curved to 20° and used to gradually pierce into the inner compact bony layer. Then, the needle was pulled out, blunted and inserted into the epidural space along the puncture site. A total of 0.2 ml autologous blood was injected into the epidural space over 5 min, and the needle was kept in the site for another 15 min to promote coagulation. The entrance of the needle was blocked with instant tissue glue and bone wax, and the incision was sutured with 3–0 silk. In the Sham group, all the procedures were performed without injecting autologous blood into the epidural space. In the Normal group, the rats were treated only with general anesthesia and subjected to blood sampling. The epidural hematoma model was confirmed by 3.0 T Magnetic Resonance Imaging (MRI) with a small round surface coil on T1 weighted imaging (WI) within 2 h. During this process, the body temperatures of the rats were kept at 37 °C (Supplementary Information Figure 1A–F).

Rats were fixed in the stereo orientation instrument and the skin was sterilized and incised down to the skull along the biparietal sutures through a single sagittal incision. A small square section of the skull (diameter 2 mm) was thinned to the inner compact bony layer on the left parietal bone 5 mm from the intersection of the sagittal suture and the arcuate suture. The syringe needle, connected with pressure manometer, was gradually penetrated into the inner bone density. All the experimental equipments in this procedure were provided by the Tianjin Key Laboratory of Cerebral Vascular and Neurodegenerative Diseases, China.

### Rabbit stress model

Rabbits were restrained in a supine position, and positive and negative electrodes for electric acupuncture were inserted to 0.3–0.5 cm at the sites 10 cm and 11 cm above the medial malleolus of the hind limb(Supplementary Information Figure 1G). The current intensity was 0.6 mA, and the frequency was 2 Hz for 10 min, and then the shock was suspended for 20 min. All the rabbits in the Restraint group were restrained for 6 h without electric shock. The Normal group animals were housed in normal conditions. To avoid unfavorable emotional irritation, all the procedures were carried out gently and quietly. The electrical stimulation instrument was provided by the Tianjin Key Laboratory of Cerebral Vascular and Neurodegenerative Diseases.

### Serum adrenocorticotropic hormone (ACTH) and growth hormone (GH) concentrations

After induction of general anesthesia with intraperitoneal administration of 0.3 ml/100 g chloral hydrate, the rats were fixed in a supine position, and blood sampling by cardiac puncture was used to sample 1 ml blood, which was injected into a coagulation-promoting tube that was centrifuged at 3,000 rpm/min for 15 min. The serum was transferred to two Eppendorf (EP) tubes and stored at −80 °C. Serum ACTH and GH levels were measured at 12 h and 24 h postoperatively with an Enzyme-Linked Immunosorbent Assay (ELISA) kit according to the manufacturer’s protocol (Cusabio Biotech Co., Wuhan, P.R. China).

### Terminal Deoxynucleotidyl Transferase (TdT)-Mediated dUTPNick End Labeling (TUNEL) Assay and immunofluorescence

Rats were anesthetized with 0.3 ml/100 g chloral hydrate administered intraperitoneally and sacrificed by perfusing the heart with 4% paraformaldehyde in Phosphate Buffered Solution (PBS) (pH 7.4). The brains were removed, postfixed overnight and sectioned by the Department of Pathology, Tianjin Huanhu Hospital. The preparation of brain sections of rabbits was same as above(Supplementary Information Figure 1H). The sections from rats were deparaffinized and rehydrated with xylene and then permeabilized with proteinase K solution. Next, the sections reacted sequentially with TdT reaction buffer and TdT reaction cocktail. The sections were then incubated with Andy Fluor™ 488-Streptavidin staining solution. Next, the sections were incubated with the primary antibody overnight at 4 °C and incubated with secondary antibodies at 37 °C for 60 min. Finally, the sections were counterstained with diamidino-phenyl-indole (DAPI) and covered with antifade mounting medium. Images were captured through fluorescence microscopy (Olympus). The sections from rabbits were tested for TUNEL according to the user manual and counterstained with hematoxylin. TUNEL kits were purchased from GeneCopoeia Inc. (rats) and Roche, USA (rabbits). DAPI and antifade mounting medium were purchased from Sigma Life Science and ZSGB-BIO Company. The primary antibodies were anti-corticotropin releasing factor (CRF) and anti-growth hormone-releasing hormone (GHRH), which were purchased from Abcam. Anti-growth hormone (GH), anti-thyroid-stimulating hormone (TSH) β were purchased from Santa Cruz Biotechnology, Inc. The secondary antibodies were Alexa Fluor® 594-Conjugated AffiniPure goat anti-mouse IgG (H + L) and Alexa Fluor® 594-conjugated AffiniPure goat anti-rabbit IgG (H + L), which were purchased from the ZSGB-BIO Company. The percentage of apoptotic cells was calculated with the formula: apoptosis rate (%) = (TUNEL-positive nuclei)/(total cell number) × 100%^26^.

### Immunohistochemistry

The sections were pretreated with xylene and then incubated with primary antibody against rat proliferating cell nuclear antigen (PCNA) or Caspas-3 (Santa Cruz Biotechnology, Inc.), at 4 °C overnight. Next, sections were incubated with complement, horseradish peroxidase (HRP) conjugate, and diazoaminobenzene (DAB), orderly (Abcam EXPOSE Mouse and Rabbit Specific HRP/DAB Detection IHC kit). Next, sections were counterstained with hematoxylin. Images were captured by a microscopy (Olympus). The images of immunostained sections were analyzed using Image Pro-Plus (IPP) software (Media Cybernetics, Silver Spring, MD, USA) to calculate the density mean, area sum, and integrated optical density (IOD) of positive expression. The PCNA and Caspase-3 levels were denoted by Average optical density (AOD). AOD = IOD(sum)/Area(sum).

### Western blot analysis

Western blot analysis was used to assess the expressions of related proteins. Specific antibodies were used to evaluate the expressions of PCNA (Santa Cruz Biotechnology), Caspase-3 (Santa Cruz Biotechnology). The tissue were treated with Radio Immunoprecipitation Assay (RIPA) Lysis Buffer (Solarbio, China) and lysed. Tissue lysates (50 μg of protein) were separated by electrophoresis 12% Sodium Dodecyl Sulfate (SDS) polyacrylamide gels and transferred to polyvinylidene difluoride (PVDF) membranes (Solarbio, China). The membranes were incubated with Tris-buffered saline (TBS) containing 1% (w/v) nonfat-milk and 0.1% (v/v) Tween-20 (TBST) for 2 h to block non-specific binding, washed with TBST for 45 min, incubated with the primary antibody at 4 °C overnight, washed with TBST for 45 min, incubated with second antibody (ZSGB-BIO Company) for 1 h, developed using enhanced chemiluminescence (ECL) (Thermo), and analyzed by Fusion Fx system (Supplementary Information Figure 2A–D).

## Results

### Intracranial hypertension of rats

**Figure 1:**
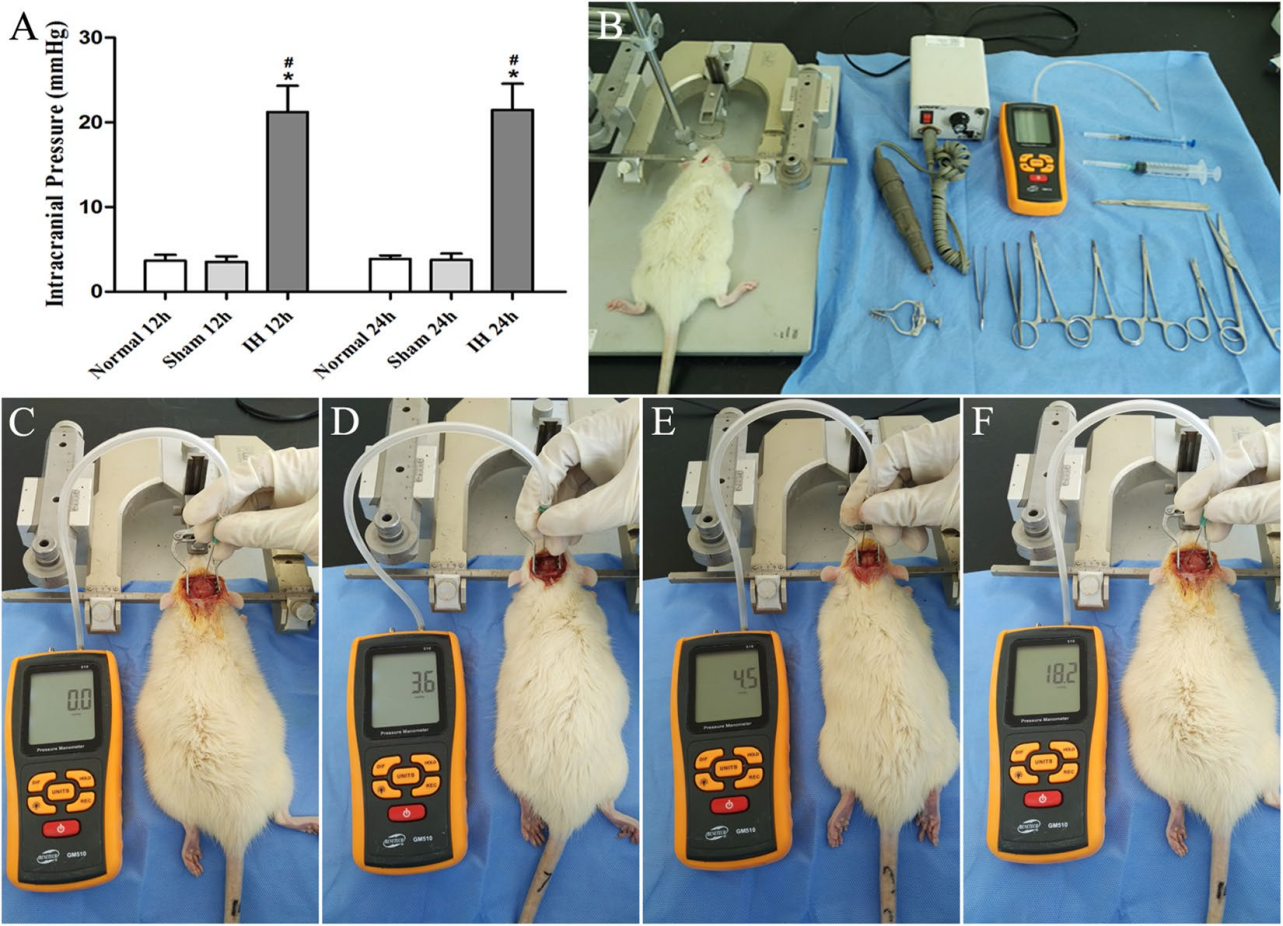
Intracranial hypertension of rat. (**A**) Shows ICP of rats. *P < 0.05 vs Normal 12 h (24 h), ^#^ *P* < 0.05 vs Sham 12 h (24 h). (**B**) Shows surgical instruments and pressure manometer. (**C**) Shows adjusted zero-position of pressure manometer. (**D**) Shows ICP in normal group 24 h. (**E**) Shows ICP in sham group 24 h. (**F**) Shows ICP in IH group 24 h.

In this study, ICP of SD rats were measured. According to the data, ICP in IH group at 12 h were significantly higher than those in the Normal group at 12 h and the Sham group at 12 h (*P* < 0.05). Furthermore, we also confirmed that ICP in IH group at 24 h also had an obvious increase than those in the Normal group and the Sham group at 24 h (*P* < 0.05). However, there was no statistical difference between IH group, Sham group, Normal group at 12 h and 24 h (*P* > 0.05) (Fig. 1).

The results demonstrated that 0.2 ml autologous blood was injected into the epidural space can increase ICP of rats. The video of measuring the ICP was provided in Dataset 1.

### The apoptosis rate and caspase-3 levels of rats’ hippocampal cells, hypothalamic and hypophyseal neuroendocrine cells at 12 h and 24 h

#### One-way ANOVA

The apoptosis rate and caspase-3 levels of rats’ hippocampal cells, hypothalamic and hypophyseal neuroendocrine cells in IH group at 12 h were significantly higher than those in the Normal group at 12 h and the Sham group at 12 h (*P* < 0.05). Moreover, the values in the Sham group at 12 h were higher than those in the Normal group at 12 h (*P* < 0.05) except AOD of caspase-3 of hypothalamic neuroendocrine cells at 12 h and 24 h (*P* > 0.05).

**Figure 2:**
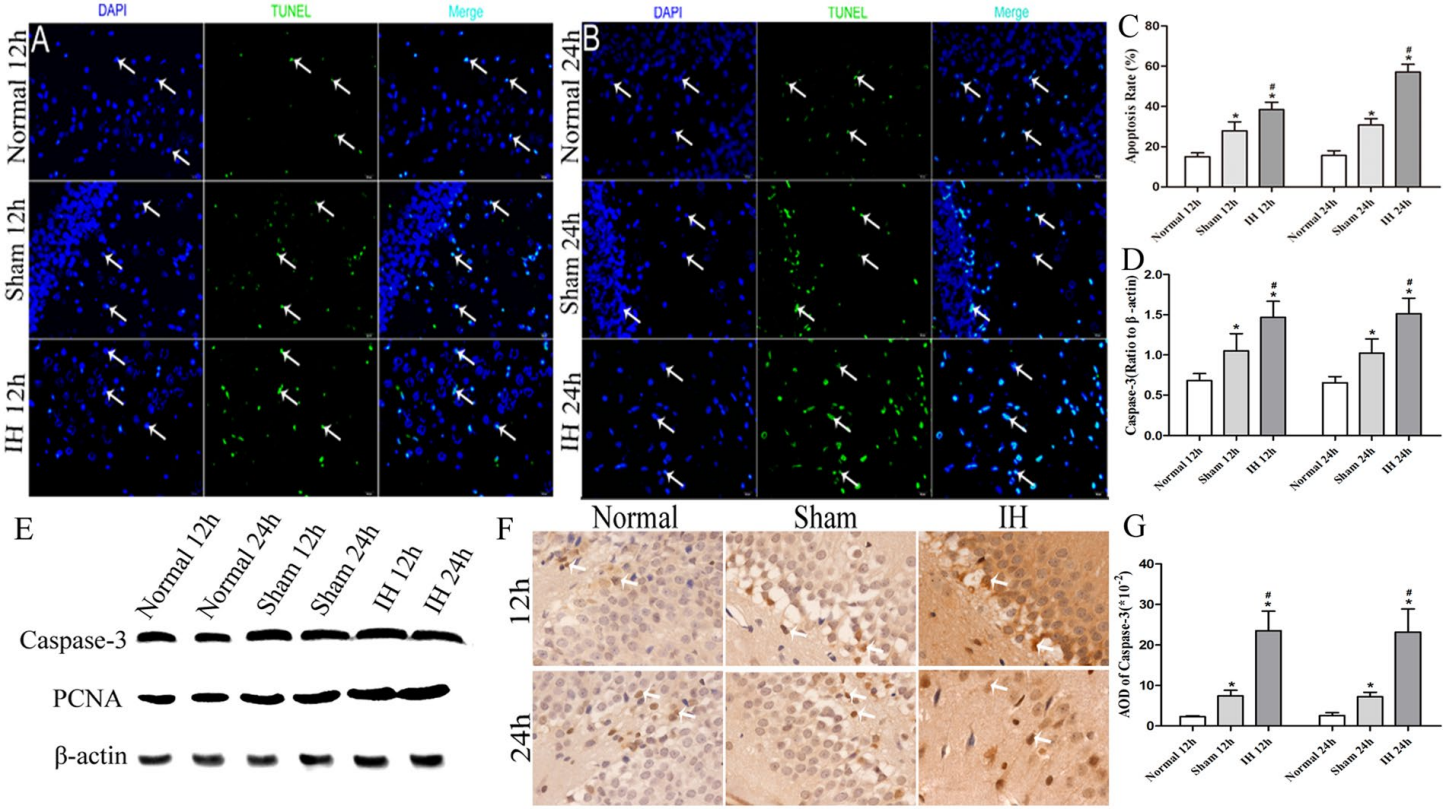
The apoptosis rate and caspase-3 levels of rats’ hippocampal cells at 12 h and 24 h. (**A**) Shows the apoptosis of hippocampal nerve cells after modeling for 12 h (**×200**). (**B**) Shows the apoptosis of hippocampal nerve cells after modeling for 24 h (**×200**). In (**A**,**B**) DAPI (blue) is used to indicate nuclei (arrows); TUNEL (green) is used to indicate apoptotic signals (arrows). Merge was used to indicate apoptotic cells (arrows). (**C**) Shows the apoptosis rate of hippocampal nerve cells after modeling for 12 h and 24 h. (**D**) Shows the Caspase-3 levels of hippocampal nerve cells after modeling for 12 h and 24 h (Western blot). (**E**) Shows the tissue lysates for protein extraction were prepared and the total protein of each lysate was equalized for Western blot analysis. Proteins were detected by specific antibodies to Caspase-3 and PCNA. β-actin was used as a loading control. (**F**) Shows immunostaining of Caspase-3 (**×400**). (**G**) Shows AOD of Caspase-3. *P < 0.05 vs Normal 12 h (24 h), ^#^ *P* < 0.05 vs Sham 12 h (24 h).

**Figure 3:**
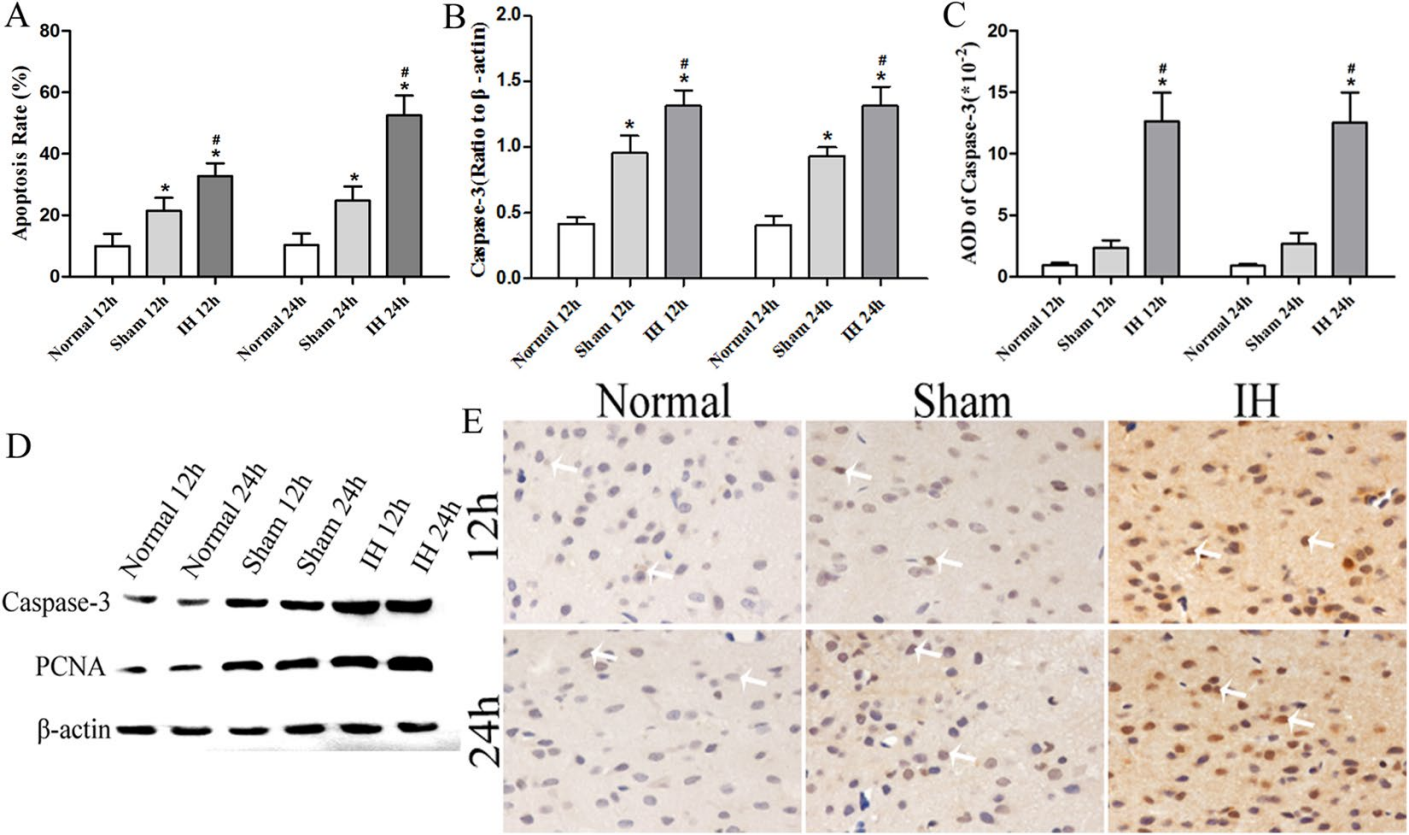
The apoptosis rate and caspase-3 levels of rats’ hypothalamic neuroendocrine cells at 12 h and 24 h. (**A**) Shows the apoptosis rate of rat hypothalamic neuroendocrine cells after modeling for 12 h and 24 h. (**B**) Shows the Caspase-3 levels of rat hypothalamic neuroendocrine cells after modeling for 12 h and 24 h (Western blot). (**C**) Shows AOD of Caspase-3. (**D**) Shows the tissue lysates for protein extraction were prepared and the total protein of each lysate was equalized for Western blot analysis. Proteins were detected by specific antibodies to Caspase-3 and PCNA. β-actin was used as a loading control. (**E**) Shows immunostaining of Caspase-3 (**×400**). *P < 0.05 vs Normal 12 h (24 h), ^#^ *P* < 0.05 vs Sham 12 h (24 h).

**Figure 4:**
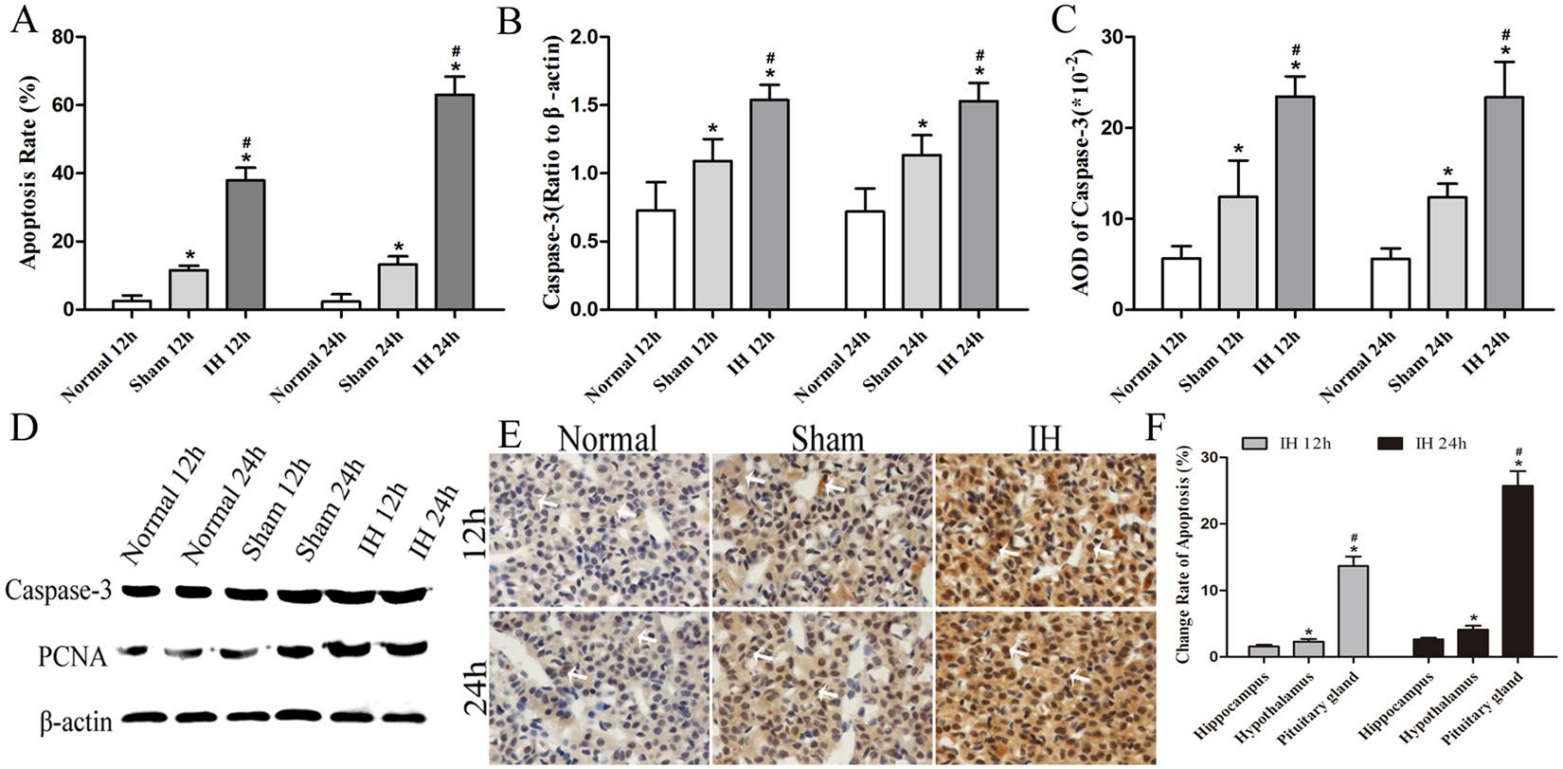
The apoptosis rates of rats’ hypophyseal neuroendocrine cells at 12 h and 24 h. (**A**) Shows the apoptosis rate of rat hypophyseal neuroendocrine cells after modeling for 12 h and 24 h. (**B**) Shows the Caspase-3 levels of rat hypophyseal neuroendocrine cells after modeling for 12 h and 24 h (Western blot). (**C**) Shows AOD of Caspase-3. (**D**) Shows the tissue lysates for protein extraction were prepared and the total protein of each lysate was equalized for Western blot analysis. Proteins were detected by specific antibodies to Caspase-3 and PCNA. β-actin was used as a loading control. (**E**) Shows immunostaining of Caspase-3 (**×400**). *P < 0.05 vs Normal 12 h (24 h), ^#^ *P* < 0.05 vs Sham 12 h (24 h). (**F**) Shows the change rate of apoptosis of the hippocampus, hypothalamus and pituitary in the intracranial hypertension group. *P < 0.05 vs hippocampus, ^#^ *P* < 0.05 vs hypothalamus.

**Figure 5:**
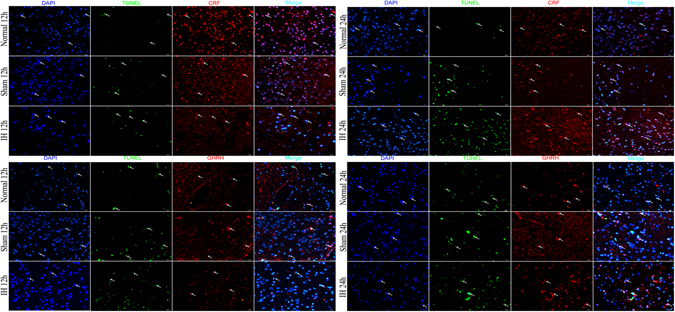
The apoptosis of rats' hypothalamic CRF/GHRH-secreted cells at 12 h and 24 h (×200). DAPI (blue) is used to indicate nuclei (arrows); TUNEL (green) is used to indicate apoptotic signals (arrows). CRF/GHRH (red) is used to indicate anti-CRF/GHRH antibodies (arrows). Merge is used to indicate apoptotic cells and CRF/GHRH (arrows).

**Figure 6:**
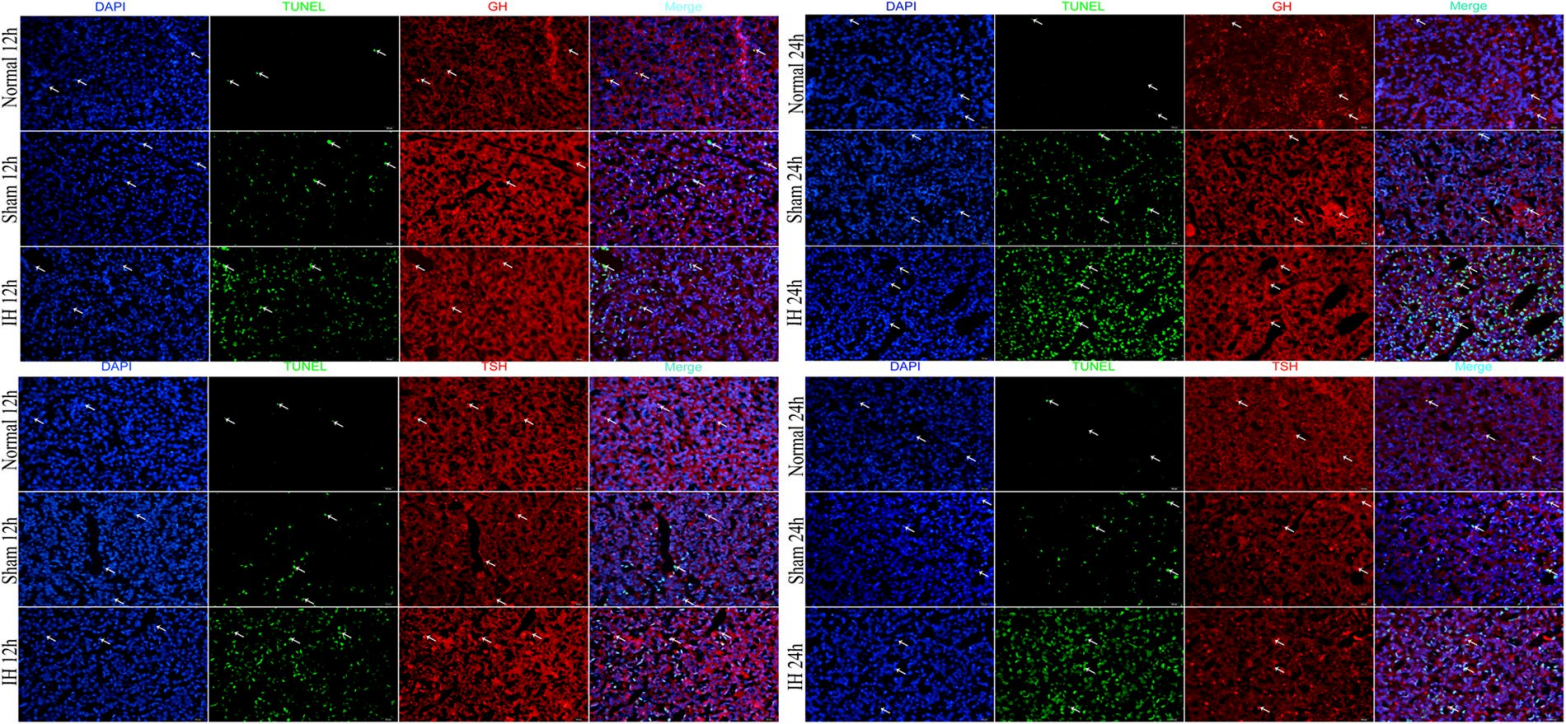
The apoptosis of rats' hypophyseal GH/TSH-secreted cells at 12 h and 24 h (×200). DAPI (blue) is used to indicate nuclei (arrows); TUNEL (green) is used to indicate apoptotic signals (arrows). GH/TSH (red) is used to indicate anti-GH/TSH antibody (arrows). Merge is used to indicate apoptotic cells and GH/TSH (arrows).

The apoptosis rate and caspase-3 levels of rats’ hippocampal cells, hypothalamic and hypophyseal neuroendocrine cells were significantly increased in the IH group at 24 h compared with Normal group and Sham group at 24 h (*P* < 0.05). The apoptosis rate and caspase-3 levels of rats’ hippocampal cells and hypophyseal neuroendocrine cells in Sham group at 24 h were higher than those in the Normal group at 24 h (*P* < 0.05). However, the apoptosis rate and caspase-3 levels of rats’ hypothalamic neuroendocrine cells in Normal group at 24 h showed no statistically significant differences from the levels in Sham group at 24 h (*P* > 0.05) (Figs 2A–G, 3A–E and 4A–E). The apoptosis of rats’ hypothalamic CRF-secreted cells and GHRH-secreted cells at 12 h and 24 h is shown in Fig. 5. The apoptosis of rats’ hypophyseal GH-secreted cells and TSH-secreted cells at 12 h and 24 h is shown in Fig. 6.

#### Independent samples t test

The apoptosis rate and caspase-3 levels of rats’ hippocampal cells, hypothalamic and hypophyseal neuroendocrine cells in Normal group at 12 h showed no statistically significant differences from the levels in the Normal group at 24 h (*P* > 0.05), and the values of Sham group at 12 h also showed no statistically significant difference compared with the Sham group at 24 h (*P* > 0.05). The apoptosis rate of rats’ hippocampal cells, hypothalamic and hypophyseal neuroendocrine cells in the IH group at 24 h were higher than those in the IH group at 12 h (*P* < 0.05). However, the caspase-3 levels of rats’ hippocampal cells, hypothalamic and hypophyseal neuroendocrine cells in the IH group at 12 h showed no statistically significant differences from the levels in the IH group at 24 h (*P* > 0.05).

The results demonstrated that the apoptosis rate and caspase-3 levels of rats’ hippocampal cells, hypothalamic and hypophyseal neuroendocrine cells were notably increased at 12 h and 24 h of intracranial hypertension. The ICP at 24 h were still increased, and the apoptosis of rats’ hippocampal cells, hypothalamic and hypophyseal neuroendocrine cells were further increased at 24 h. The results indicated that the apoptosis rate at 24 h was further increased in the case of intracranial hypertension at 12 h and 24 h. We also found that craniotomy can lead to apoptosis of hippocampal nerve cells, hypothalamic and hypophyseal neuroendocrine cells as compared with results of normal rats. Moreover, the craniotomy-induced apoptosis of hippocampal nerve cells, hypothalamic and hypophyseal neuroendocrine cells showed no differences between 12 h and 24 h.

### Changing rates of apoptosis in the hippocampus, hypothalamus and pituitary gland in the IH group

#### One-way ANOVA

In the IH group at 12 h and 24 h, compared with the change rates of apoptosis in the hippocampus, the change rates of apoptosis in the hypothalamus and pituitary gland were significantly higher (*P* < 0.05) (Fig. 4F). Moreover, the change rate of apoptosis in the pituitary gland was significantly higher than that in the hypothalamus (*P* < 0.05) (Fig. 4F). These results demonstrated that increased intracranial pressure leads to apoptosis of the hippocampal, hypothalamic and hypophyseal cells. Furthermore, the change rate of apoptosis in the pituitary gland was the largest, thus indicating that the pituitary gland is the most sensitive to increased intracranial pressure, followed by the hypothalamus and hippocampus.

### Relative PCNA levels of the hippocampus, hypothalamus and pituitary gland in rats

**Figure 7:**
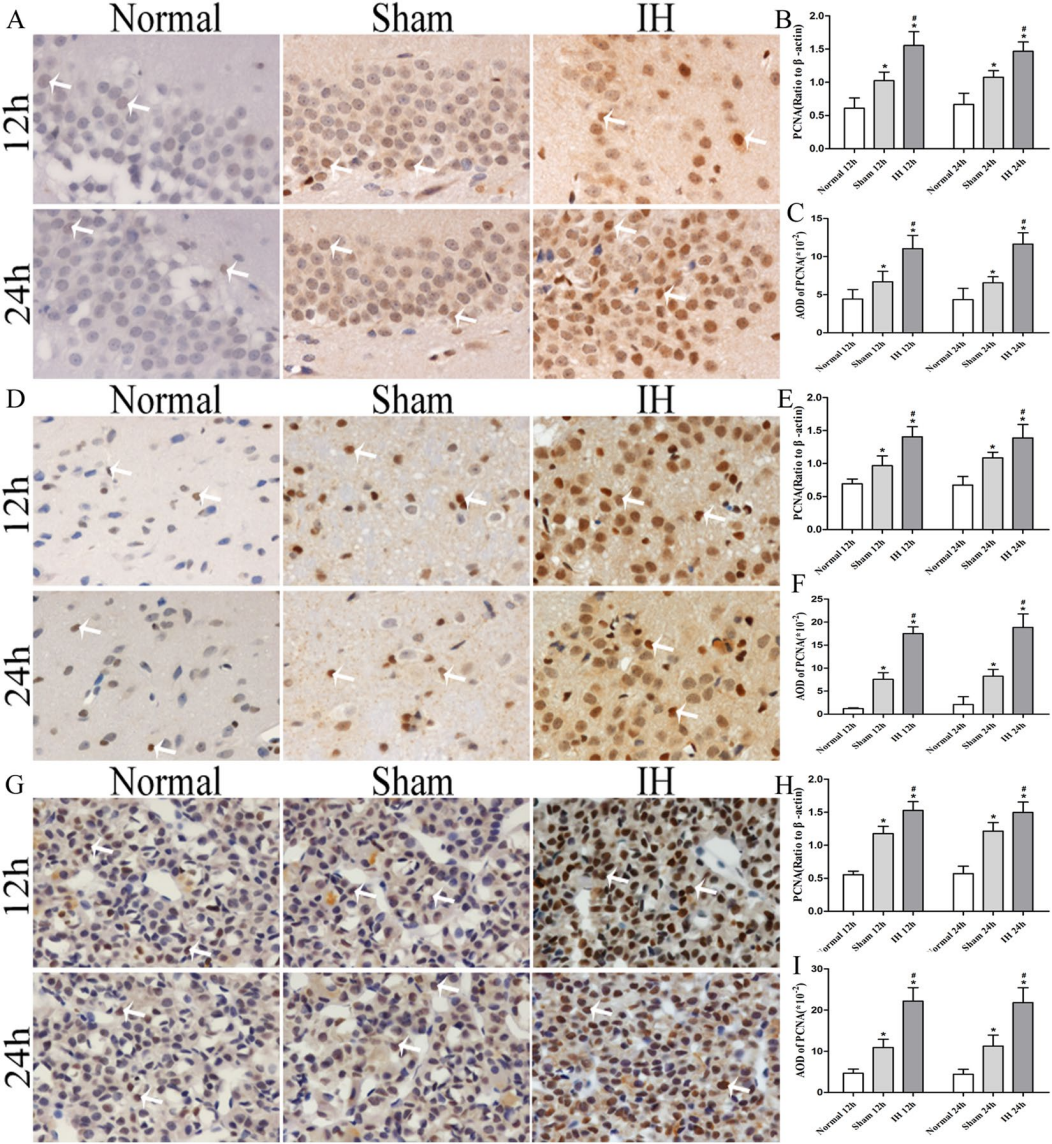
The PCNA levels of the hippocampus, hypothalamus and pituitary gland in rats. (**A**,**D**,**G**) Show immunostaining of PCNA (**×400**). (**B**,**E**,**H**) Show the PCNA levels of the hippocampus, hypothalamus and pituitary gland in rats after modeling for 12 h and 24 h (Western blot). (**C**,**F**,**I**) Show AOD of PCNA in the hippocampus, hypothalamus and pituitary gland of rats after modeling at 12h and 24h. *P < 0.05 vs Normal 12 h (24 h), ^#^ *P* < 0.05 vs Sham 12 h (24 h).

The relative PCNA levels showed a consistent trend in the rats’ hippocampus, hypothalamus and pituitary gland. The relative PCNA levels were the highest in the IH group at 12 h/24 h compared with the Normal group at 12 h/24 h and the Sham group at 12 h/24 h (*P* < 0.05). Moreover, compared with the Normal group at 12 h/24 h, the Sham group at 12 h/24 h had increased relative PCNA levels (*P* < 0.05). However, the relative PCNA levels of the hippocampus, hypothalamus and pituitary gland in rats showed no differences between the 12 h group and the 24 h group (*P* > 0.05) (Figs 2E, 3D, 4D and 7A–I).

### Rat serum GH (ng/ml) and ACTH (pg/ml) concentrations at 12 h and 24 h

#### One-way ANOVA

**Figure 8:**
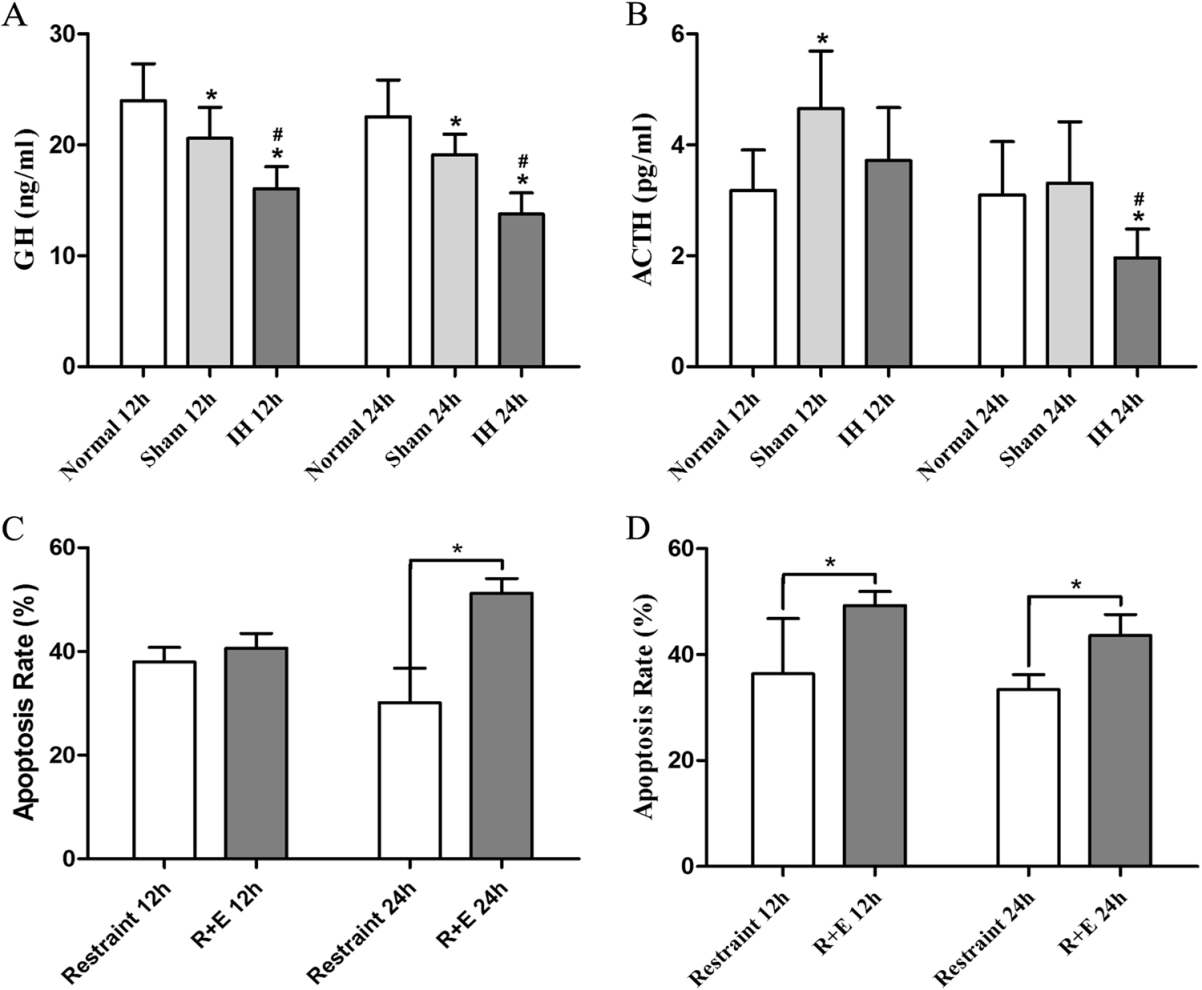
The rat serum GH and ACTH concentrations and the apoptosis rates of rabbit hypothalamic and hypophyseal neuroendocrine cells at 12 h and 24 h. (**A**) Shows GH concentrations after modeling for 12 h and 24 h. (**B**) Shows ACTH concentrations after modeling for 12 h and 24 h. *P < 0.05 vs Normal 12 h (24 h), ^#^ *P*  < 0.05 vs Sham 12 h (24 h). *P < 0.05. (**C**) Shows the apoptosis rate of rabbit hypothalamic neuroendocrine cells at 12 h and 24 h. The apoptosis rate of the hypothalamus in the Normal group was 0%. (**D**) Shows the apoptosis rate of rabbit hypophyseal neuroendocrine cells at 12 h and 24 h. The apoptosis rate of hypophyseal neuroendocrine cells in Normal group was 0%. *P < 0.05.

The GH concentration was the lowest in the IH group at 12 h compared with the Normal group at 12 h and the Sham group at 12 h (*P* < 0.05) (Fig. 8A). Moreover, compared with the Normal group at 12 h, in the Sham group at 12 h, the GH concentration was decreased (*P* < 0.05) (Fig. 8A). Compared with the levels in the Normal group at 24 h and the Sham group at 24 h, the GH concentration in the IH group at 24 h was decreased significantly (*P* < 0.05) (Fig. 8A).

#### Independent samples t test

The GH concentration in the IH group at 24 h was lower than that in the IH group at 12 h (*P* < 0.05).

The results demonstrated that the concentration of GH at 24 h was further dropped in the case of intracranial hypertension at 12 h and 24 h. Craniotomy was found to decrease serum GH concentrations.

#### One-way ANOVA

The ACTH concentrations were statistically significantly different between the Normal group at 12 h and the Sham group at 12 h (*P* < 0.05) (Fig. 8B). The Normal group at 12 h showed no differences compared with the IH group at 12 h (*P* > 0.05) (Fig. 8B). Compared with the concentrations in the Normal group at 24 h and the Sham group at 24 h, the serum ACTH concentration in the IH group at 24 h was decreased markedly (*P* < 0.05) (Fig. 8B). Moreover, the serum ACTH concentrations did not differ between the Normal group at 24 h and the Sham group at 24 h (*P* > 0.05) (Fig. 8B).

#### Independent samples t test

The ACTH concentration in IH group at 24 h was lower than that in the IH group at 12 h (*P* < 0.05).

These results demonstrate that the serum ACTH concentration was increased at 12 h after craniotomy and was induced by stress. However, the serum ACTH concentration was maintained at a Normal level at 12 h in the case of intracranial hypertension. This observation suggested that ACTH-secreting cells in the IH group at 12 h underwent apoptosis. The serum ACTH concentration was maintained at a Normal level 24 h after craniotomy, and these results indicated that apoptosis of neuroendocrine cells induced by craniotomy was sufficient to compensate for the normal serum ACTH concentration. However, the serum ACTH concentration in the IH group at 24 h was significantly lower than the concentration in the Normal group. This result indicated that the concentration of ACTH at 24 h was further reduced in the case of intracranial hypertension at 12 h and 24 h.

### The apoptosis rate of rabbit hypothalamic and hypophyseal neuroendocrine cells at 12 h and 24 h

#### Independent sample t test

The apoptosis rate of the hypothalamus in the Normal group at 12 h and 24 h was 0%. There were no statistically significant differences in the apoptosis rates of hypothalamic neuroendocrine cells between the Restraint group at 12 h and the R + E group at 12 h (*P* > 0.05) (Fig. 8C). Compared with the Restraint group at 24 h, in the R + E group at 24 h, the apoptosis rate of hypothalamic neuroendocrine cells was increased (*P* < 0.05) (Fig. 8C). These results confirmed that stress leads to apoptosis of neuroendocrine cells in the hypothalamus. Moreover, the stress intensity was positively correlated with the apoptosis rate in the hypothalamus.

#### Independent sample t test

**Figure 9:**
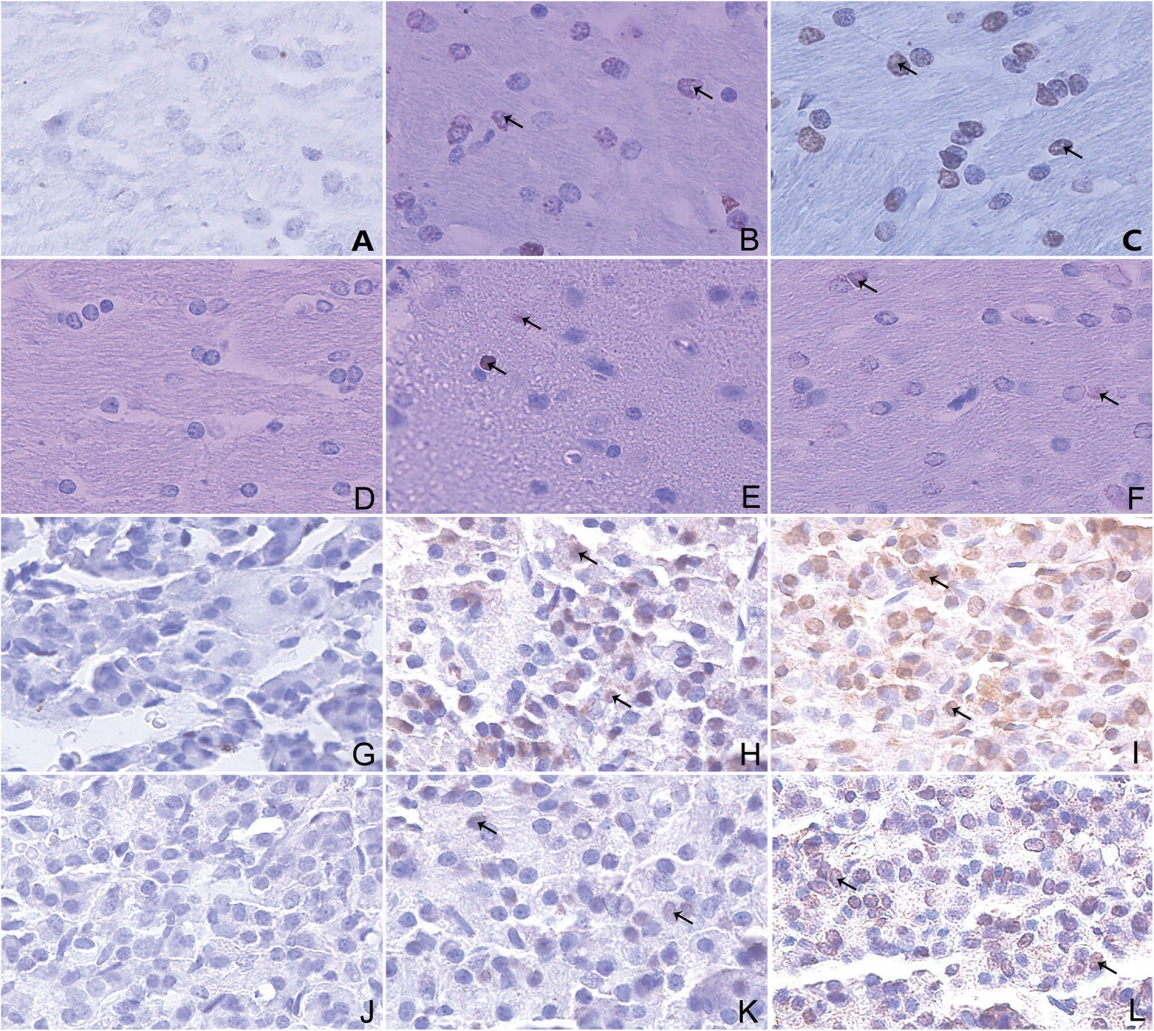
The immunohistochemistry images show apoptosis of rabbit hypothalamic and hypophyseal neuroendocrine cells at 12 h and 24 h (×400). (**A**–**F**) Show the apoptosis of rabbit hypothalamic neuroendocrine cells at 12 h and 24 h. (**G**–**L**) Show the apoptosis of rabbit hypophyseal neuroendocrine cells at 12 h and 24 h. (**A**,**G**) Show Normal 12 h. (**B**,**H**) Show Restraint 12 h. (**C**,**I**) Show R + E group 12 h. (**D**,**J**) Show Normal 24 h. (**E**,**K**) Show Restraint 24 h. (**F**,**L**) Show R + E 24 h. The arrows in the panels show the apoptotic cells.

The apoptosis rate of pituitary gland in Normal group 12 h and 24 h was 0%. The apoptosis rate of pituitary gland in R + E group 12 h were higher than Restraint group 12 h (*P* < 0.05) (Fig. 8D). Compared with Restraint group 24 h, the apoptosis rate of pituitary gland was increased in R + E group 24 h (*P *< 0.05) (Fig. 8D). The results confirmed that stress can lead to apoptosis of neuroendocrine cell in pituitary gland, moreover, the stress intensity was positive correlated with the apoptosis rate of pituitary gland. The apoptosis rates of rabbit hypothalamic neuroendocrine cells at 12 h and 24 h are shown in Fig. 9A–F. The apoptosis of rabbits’ hypophyseal neuroendocrine cells at 12 h and 24 h were showed in Fig. 9G–L.

## Discussion

Through the establishment of an epidural hematoma model, we studied the effects of increased intracranial pressure on the apoptosis rates of hippocampal, hypothalamic and hypophyseal neuroendocrine cells. In our study, 0.2 ml autologous blood was injected into the epidural space to establish an epidural hematoma and induce intracranial hypertension in the experimental group with SD rats (body weight 300–320 g). We measured rats’ ICP at 12 h and 24 h after the establishment of epidural hematoma model. According to the data, we confirmed that ICP increased at 12 h and 24 h in the animal model.

The hippocampus is crucial for human learning and memory. Moreover, except for focal lesions of the cortex, the hippocampus is the area that is most easily damaged by TBI^27^. Therefore, it was very interesting to explore the apoptosis rates of hippocampal cells caused by intracranial hypertension. Conti *et al*. have also found that the apoptosis rate of the hippocampus is most obvious in the adult rat brain at 12-48 h after establishment of a lateral fluid-percussion (FP) brain injury model^28^. Our experimental results showed that the apoptosis rate of hippocampal cells was significantly increased when intracranial hypertension was confirmed at 12 h, and the apoptosis rate of hippocampal cells was further increased in the case of intracranial hypertension at 24 h. Moreover, intracranial hypertension also induced the apoptosis of hypothalamic neuroendocrine cells and hypophyseal neuroendocrine cells. The tendency of apoptosis in the hypothalamus and pituitary gland was similar to that of hippocampus. Under the same intracranial hypertension conditions, the change rate of apoptosis in the pituitary gland was larger than those in the hypothalamus and hippocampus. Furthermore, the change rate of apoptosis in the hypothalamus was greater than that in the hippocampus. Therefore, we believed that the pituitary gland is the most sensitive to increases in intracranial pressure, followed by the hypothalamus and hippocampus. However, we also found that craniotomy itself led to the apoptosis of hippocampal cells, hypothalamic neuroendocrine cells and hypophyseal neuroendocrine cells. Moreover, the craniotomy-induced apoptosis rate of the cells did not differ between 12 h and 24 h. Therefore, it suggested that the craniotomy itself is a source of stress for the animals, which may be an independent risk factor for apoptosis. We also examined the expression profiles of PCNA and active caspase-3, whose changes correlated with the apoptosis rate except the differences between 12 h and 24 h.

Giannoudis *et al*. have reported that surgical stress is the effect of an operation on the human body, and the severity of stress is proportional to the degree of tissue damage^29^. According to previous studies, TBI, serving as a stressor, has been found to alter the hypothalamic-pituitary-adrenal (HPA) axis^30–32^. Furthermore, the HPA axis serves as a key adaptive system addressing various stresses^33^. ACTH-secreting cells are representative of the HPA axis, which responds to various conditions by controlling adrenocortical functions. Therefore, activation of the HPA axis leads to the release of ACTH^34^. Additionally, the serum ACTH concentration served as a proxy for the degree of stress. In our research, the ACTH concentration was notably increased by the sham craniotomy operation, and the ACTH concentration was restored to normal levels 24 h after the sham craniotomy operation. This result demonstrated that the hypothalamic and hypophyseal neuroendocrine cells were sufficient to compensate for the apoptosis induced by surgical stress. After the stress was stopped, the factors of apoptosis also gradually decreased. The serum ACTH concentration was not significantly increased at 12 h in the situation of intracranial hypertension, and the serum ACTH concentration was significantly lower than the normal level at 24 h in the circumstance of intracranial hypertension. The severe apoptosis of the hypothalamic and hypophyseal neuroendocrine cells caused by intracranial hypertension was presumed to be the reason for the low ACTH concentrations.

Apoptosis of the nerve cells in the rats' Sham group may be an occasional phenomenon or may be induced by interspecies diversity. To further verify that apoptosis was induced by stress in rats, and to eliminate the diversity of species, we chose rabbits to carry out a study on stress, which was different from stressed caused by an operation. Therefore, to explore the effect of stress on the apoptosis of hypothalamic and hypophyseal neuroendocrine cells, we used an animal stress model that is widely accepted. Moreover, restraint and electrical stimulation were used to increase the intensity of stress. The results confirmed that stress led to apoptosis of neuroendocrine cells in the hypothalamus and pituitary gland. Moreover, the higher the stress intensity, the higher the apoptosis rate in the hypothalamus and pituitary gland.

Prospective clinical studies have found that serum GH is the most commonly reduced hormone in the acute phase of TBI^35^. A large number of studies have reported that GH is the most common hormone in the dysfunction of the hypothalamic-pituitary axis^8, 36, 37^. Therefore, we chose the GH concentration as a readout for monitoring activities. Our results demonstrated that the serum GH concentration was significantly decreased at 12 h when the ICP increased, and the serum GH concentration was further decreased at 24 h under the circumstance of intracranial hypertension. These results were in line with the TUNEL results; that is, the apoptosis rates of the hypothalamus and pituitary gland were significantly higher at 24 h than 12 h after the intracranial hypertension treatment.

Through analysis of the change in ACTH and GH concentrations, we found that surgical stress increased the concentration of ACTH and decreased the concentration of GH after 12 h. The concentration of ACTH recovered to normal levels 24 h after surgical stress, however, the concentration of GH decreased continuously. The ACTH concentration remained at normal levels, and the GH concentration decreased at 12 h in the case of intracranial hypertension, and the levels of both decreased at 24 h in which time the intracranial pressure increased. A possible reason for this phenomenon is that ACTH cells are regulated by autonomic nerves and the endocrine system, and their regulatory mechanism is more complex than that of GH cells. In addition, GH cells may be more sensitive to stress than ACTH cells, and this phenomenon still requires further study.

## Electronic supplementary material

Dataset 1

Supplementary Information
