# Supplementary material for: Assessment of the role of intracranial hypertension and stress on hippocampal cell apoptosis and hypothalamic-pituitary dysfunction after TBI
Source: Sci Rep. 2017 Jun 19;7:3805. doi: 10.1038/s41598-017-04008-w (PMC5476648; doi:10.1038/s41598-017-04008-w)
Supplement: Supplementary file 2 — Supplementary Information [file 41598_2017_4008_MOESM2_ESM.pdf]

Assessment of the role of intracranial hypertension and stress on hippocampal cell apoptosis and hypothalamic-pituitary dysfunction after TBI

Huajun Tan [ab], Weijian Yang [ab], Chenggang Wu [c], Baolong Liu [d], Hao Lu [a] , Hong Wang [b,*], Hua Yan [b, e,*]

a Graduate School of Tianjin Medical University, Tianjin 300070, China.

b Department of Neurosurgery, Tianjin Huanhu Hospital, Tianjin 300350, China

c Department of Neurosurgery, Second Affiliated Hospital of Tianjin University of Traditional Chinese Medicine, Tianjin 300151, China.

d Department of Ultrasonography, Tianjin Huanhu Hospital, Tianjin 300350, China.

e Tianjin Key Laboratory of Cerebral Vascular and Neurodegenerative Diseases, Tianjin 300350, China.

[*] Correspondence and requests for materials should be addressed to Hua Yan (yanhua20042007@sina.com).

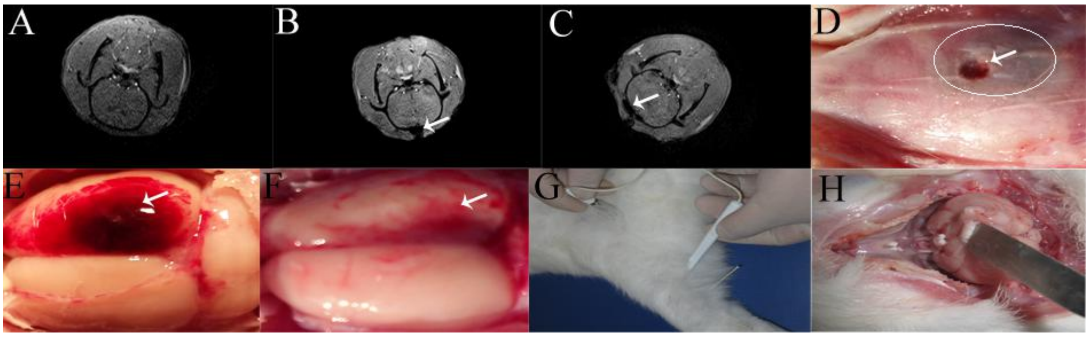

Fig. 1 Rat epidural hematoma model, rabbit stress model and brain tissue samples. Rat epidural hematoma (A-F), rabbit stress model and brain tissue samples (G-H). (A), (B), and (C) show 3.0T MRI T1WI. (A) Shows a normal rat. (B) Shows a sham rat bone window (arrow). (C) Shows an epidural hematoma (arrow). (D) Shows the bone window of an epidural hematoma rat (arrow). The area of the epidural hematoma is marked by a white line, and the dark red hematoma can be observed through the skull (E) Shows an epidural hematoma (arrow). (F) Shows that the epidural hematoma had cleared. (G) Shows the electric acupuncture insertion location. (H) Shows brain tissue samples.

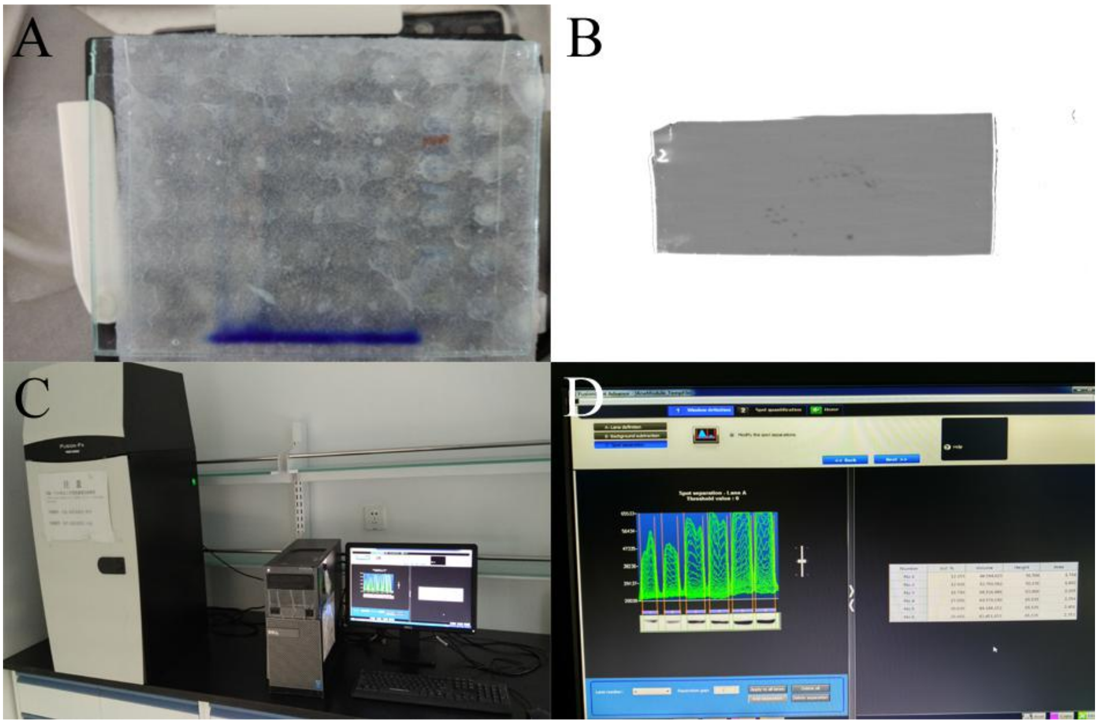

Fig. 2 Gel and Fusion Fx system. (A) shows 12% SDS polyacrylamide gel. (B) shows PVDF membrane. (C) shows the hardware of Fusion Fx system. (D) shows the software of Fusion Fx system.

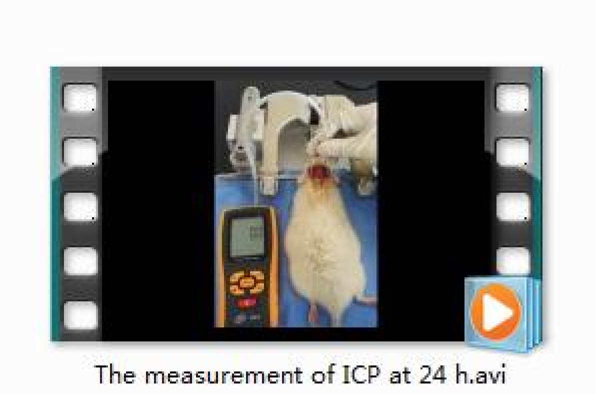

Video The measurement of ICP at 24 h
